# Fœtal Malaria

**Published:** 1890-01-25

**Authors:** 


### FŒTAL MALARIA.

Dr. Felkin, lecturer on diseases of the tropics and climatology, Edinburgh School of Medicine, has contributed a paper headed "Fœtal Malaria" (*Edinburgh Medical Journal*). He remarks on the recognised fact that a *fœtus in utero* may suffer from a paroxysm of malarial fever, and be born with ague cake, and records two instances in which fœtal ague occurred. In the first case he could feel the fœtus shaking, although the mother was not affected, excepting from the fright caused by the curious sensations in the abdomen, and had never had ague, and never suffered from malaria in any form whatever. The husband had been employed for several years on the West Coast of Africa, and had suffered severely from both intermittent and remittent, so much so that he decided to throw up his employment, and to try a change of climate to the Cape after a holiday in Madeira, where he met his future wife. She was a Lancashire lady, and they remained for eight months in Madeira, arriving at Durban, in South Africa, a week before Dr. Felkin was called in. A few days afterwards the child was born with enlarged spleen. Dr. Felkin observes that if this case had occurred alone, it might be supposed that the mother had in some way or other have become infected by malaria, but his next case he believes precludes all such possibility. In 1888 Dr. Felkin was called to see a pregnant female who complained of "fluttering in the abdomen," lasting about an hour, which recurred at intervals. The child was born with large spleen, and had two attacks of ague within 48 hours of its birth, from the latter of which attacks it died. The child's parents had been married twelve years. The mother had never been away from Edinburgh, but the father had been to West Indian ports, and, like the first husband, suffered from severe remittent.

Now, it is admitted that the fœtus may suffer from syphilis transmitted by the father, the mother remaining well. The virus of disease is probably as specific and individual as are the seeds of barley or clover. If it is transmitted to offspring through the sperm or germ, then the fœtus is liable to the full development of the disease, and if it chance that none of its elements do so pass, then the offspring, although born to a tainted parent, escapes free. There seems no reason why if one disease is transmitted, another malady should not be transmitted also.

